# Effects of Hyponatremia on the Brain

**DOI:** 10.3390/jcm3041163

**Published:** 2014-10-28

**Authors:** Corinna Giuliani, Alessandro Peri

**Affiliations:** Endocrine Unit, Department of Experimental and Clinical Biomedical Sciences “Mario Serio”, University of Florence, Florence 50139, Italy; E-Mail: corinna.giuliani@tiscali.it

**Keywords:** hyponatremia, hyponatremic encephalopathy, osmotic demyelination syndrome, brain

## Abstract

Hyponatremia is a very common electrolyte disorder, especially in the elderly, and is associated with significant morbidity, mortality and disability. In particular, the consequences of acute hyponatremia on the brain may be severe, including permanent disability and death. Also chronic hyponatremia can affect the health status, causing attention deficit, gait instability, increased risk of falls and fractures, and osteoporosis. Furthermore, an overly rapid correction of hyponatremia can be associated with irreversible brain damage, which may be the result of the osmotic demyelination syndrome. This review analyzes the detrimental consequences of acute and chronic hyponatremia and its inappropriate correction on the brain and the underlying physiopathological mechanisms, with a particular attention to the less known *in vivo* and *in vitro* effects of chronic hyponatremia.

## 1. Introduction

Hyponatremia, defined as a serum sodium concentration ([Na^+^]) below 136 mEq/L, is the most common electrolyte abnormality in hospitalized patients [1] and is associated with significant morbidity and mortality [2,3]. The link between hyponatremia and brain is strong and mutual; in fact several neurologic diseases frequently are associated with hyponatremia and hyponatremia itself causes serious clinical consequences that involve the central nervous system [4]. Therefore, it is not surprising that hyponatremia is very frequently encountered in neurosurgical and neurocritical care settings, where it is present in up to 50% and 38% of patients, respectively [1,4]. Common neurological pathologies, including subarachnoid haemorrhage, cerebrovascular accidents, brain tumour and head trauma [5,6,7], result in hyponatremia secondary to the syndrome of inappropriate secretion of antidiuretic hormone (SIADH) or to the cerebral salt wasting syndrome (CSW), which are a consequence of the release of ADH or natriuretic peptides, respectively, from the brain as a response to an injury. Furthermore, several drugs acting on the nervous system and frequently used in neurosurgical or neurologic/psychiatric patients, *i.e.*, antidepressants and antiepileptic drugs, may cause hyponatremia secondary to SIADH.

The clinical manifestations of hyponatremia are especially related to dysfunction of the central nervous system, and they are more dramatic when a marked decrease of serum [Na^+^] occurs acutely [7]. Hyponatremic encephalopathy is a well-known consequence of the brain swelling secondary to acute hyponatremia and is associated with an overall mortality of 34% [8]. Chronic hyponatremia is associated to adaptive responses counteracting swelling in brain cells and is traditionally defined as an asymptomatic or pauci-symptomatic condition [7,9]. However, there is recent evidence that also chronic and mild hyponatremia is associated with significant clinical consequences involving especially the bone and the central nervous system [2,10]. Furthermore, the correction of hyponatremia, when inappropriate, could be associated with irreversible brain damage [7].

The present review analyzes the effects of acute and chronic hyponatremia and its correction on the central nervous system and clarifies the underlying mechanisms leading to brain injury.

## 2. Pathophysiology of Brain Swelling and Adaptive Response in Hyponatremia: The “Osmotic Theory”

**Figure 1:**
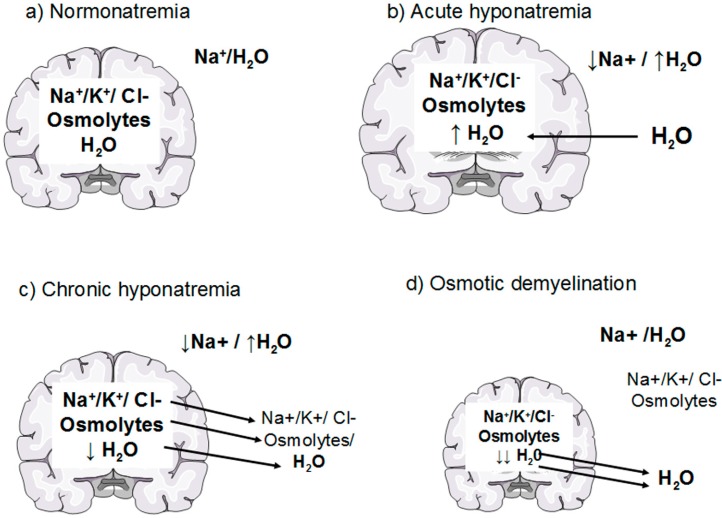
Effects of hyponatremia on the brain and adaptive mechanism. (**a**) Normonatremia: brain osmolality is in equilibrium with extracellular fluid osmolality; (**b**) Acute hyponatremia: water moves into the brain in response to an osmotic gradient, causing brain swelling; (**c**) Chronic hyponatremia: within a few hours cells loss electrolytes (rapid adaption) and later on organic osmolytes (slow adaption); the consequent loss of osmotically obligated water reduces cellular swelling and normalizes brain volume (**d**) Osmotic demyelination: an overly rapid correction of hyponatremia causes an inverse osmotic gradient with an excessive loss of water from the cells causing brain dehydration and demyelination of white matter.

**Figure 2:**
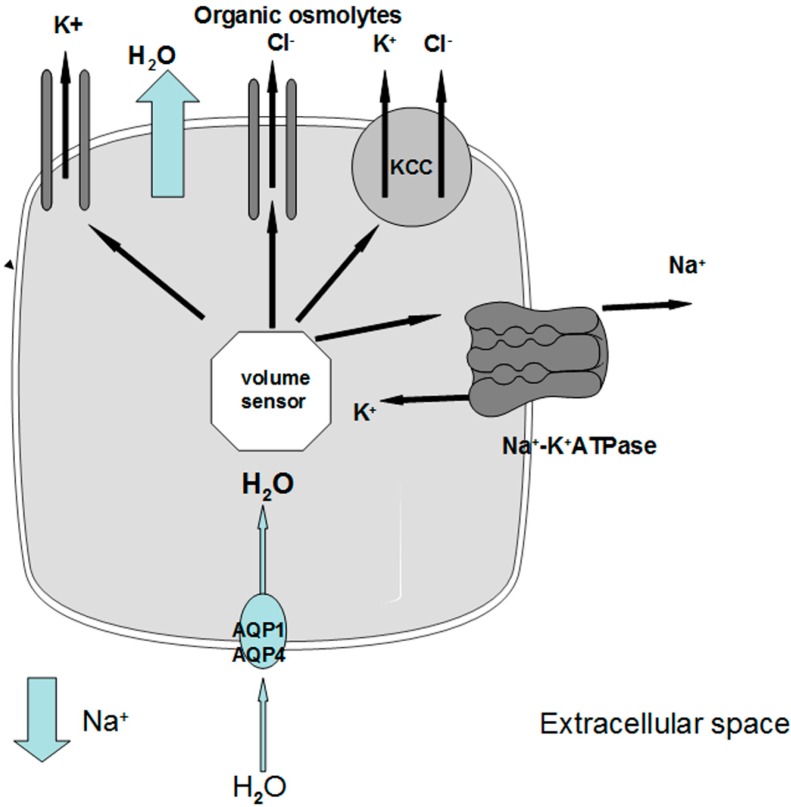
Schematic representation of cellular responses leading to swelling in chronic hyponatremia. When extracellular [Na^+^] decreases, water entry into the cells through AQP1 and AQP4 causes an increase in cell volume that triggers a volume sensor. Activation of the volume sensor causes an efflux of electrolytes through the Na^+^-K^+^ ATPase, the K^+^ channels, the KCC transporter and a volume sensitive Cl^−^ channel that also mediates the extrusion of Cl^−^ and organic osmolytes. AQP1: type 1 aquaporin water channel; AQP4: type 4 aquaporin water channel; KCC: K^+^-Cl^−^ cotransporter.

Under physiological conditions, brain osmolality is in equilibrium with extracellular fluid osmolality (Figure 1a). When hyponatremia occurs, the resulting decrease in plasma osmolality (with the exception of the rare cases of non-hypoosmotic hyponatremia) causes water movement into the brain in response to the osmotic gradient, thus causing cerebral edema [7,8] (Figure 1b). The cells most involved in swelling are the astocytes, a kind of glial cells that are a constituent of the blood-brain barrier and have a fundamental role in maintaining the fluid and electrolyte concentration of the extracellular space in the brain [11]. Glial cells selectively swell in presence of hyposmolar stress with sparing of neurons, suggesting the presence of specific water channels localised in astrocytes to protect neurons from the water entry [11,12]. Recent evidence has suggested the presence of aquaporin (AQP) water channels in the glia, in particular AQP1 and AQP4 subtypes, which appear to be important in the development of cerebral edema during hyponatremia [13]. In fact, when hypoosmolality occurs, water moves through AQP1 and AQP4 channels into the glial cells, which selectively swell whereas neurons are relatively spared [14,15]. Nevertheless, the glial cells are not perfectly osmometers and an initial swelling of the brain occurs. The presence of adaptive mechanisms counteracting brain swelling represents an ancient conserved homeostatic response that is essential for the cell survival, because cell volume alterations can change cell functions such as cell-cycle progression, proliferation, apoptosis, excitability and metabolism [16]. The regulation of cell volume is well documented in the brain, in which the physical restriction of the skull limits the expansion and makes essential the presence of accurate adaptive mechanism counteracting brain swelling. The first adaptive response is a compensatory displacement of fluid from the interstitial space into the cerebrospinal fluid and from there into the systemic circulation [17]. The next and more sustained adaptive mechanism, known as “volume regulatory decrease” (VRD) [17,18], is the extrusion of intracellular solutes together with osmotically obligated water to reduce cellular swelling and normalize brain volume (Figure 1c). How the cells sense the volume increase and which transduction pathways are involved remain to be elucidated, but a hypothetical membrane receptor with an intrinsic tyrosine kinase activity could be implicated [17]. During the first three hours, cells mainly lose inorganic ions, such as Na^+^, K^+^ and Cl^−^. The first pathway activated by brain swelling is the energy-dependent extrusion of Na^+^ by the Na^+^-K^+^ ATPase pump [8], which represents the main defence against cerebral edema. Subsequently, other osmotically active ions are extruded via cellular channels including Ca^++^-dependent and -independent K^+^ channels, the K^+^-Cl^−^ co-transporter and the volume-sensitive Cl^−^ channel [8,9,17,18,19]. This mechanism accounts for 65% of the observed brain volume regulation [9,17]. A second response consists of the loss of small organic osmolytes, in particular amino acids (glutamate, taurine, glycine) and myo-inositol through a putative volume-sensitive organic osmolyte and anion channel [9,17]. The latter is probably a swelling-activated Cl^−^ channel that also mediates the extrusion of organic osmotically active solutes [17] (Figure 2). The efflux of organic osmolytes is sustained as long as hyponatremia persists, becoming an essential adaptive mechanism in chronic hyponatremia. The contribution of organic osmolytes to the brain volume regulation has been estimated as 35% [17]. It has to be said that several of the organic osmolytes lost, in particular glutamate, are neuroactive and therefore could produce transient neurological abnormalities, such as increased seizures activity [9,18] and decreased synaptic release of excitatory neurotransmitters, which could explain the gait instability observed in chronically hyponatremic patients [10,18].

The process of brain volume regulation is of fundamental importance in order to understand the variability of the clinical presentation of hyponatremia. When hypoosmolality arises at a rate that exceeds the brain ability to regulate its volume by electrolyte losses, such as in acute hyponatremia (<48 hours), brain edema may occur and the patients can develop severe neurological signs and symptoms, possibly leading to death for brain herniation [7,20]. In chronic hyponatremia, in which the imbalance is present by more than 48 h, the loss of both electrolyte and organic osmolytes represents a very efficient mechanism to regulate brain volume. Therefore, in chronic hyponatremia brain edema is minimized and neurological symptoms may be absent or mild [7,21].

**Figure 3:**
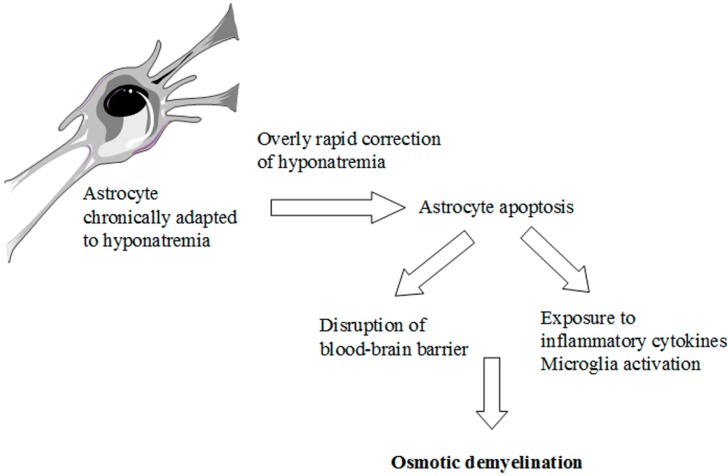
Pathophysiology of ODS. Schematic representation of the possible events caused by an overly rapid correction of chronic hyponatremia.

The knowledge of these complex adaptive mechanisms is very important when we correct hyponatremia with an active therapy. Here, a reversal of the compensatory responses secondary to hyponatremia occurs [2,7,9]. In particular, recapture of organic osmolytes is needed, in order to prevent a fluid loss from cells. This is a very slow process, which may take several days [18,22,23]. Therefore, an overly rapid correction of chronic hyponatremia, that exceeds the brain’s ability to recapture the lost osmolytes, causes an inverse osmotic gradient with a consequent dehydration of brain tissue and possible demyelination of the white matter [7] (Figure 1d). This dramatic consequence is known as osmotic demyelination syndrome (ODS) [24] and occurs especially in the pons (central pontine myelinolysis), although an extrapontine myelinolysis affecting the basal ganglia, cortex, lateral geniculate body and internal capsule can also occur [25]. Also in the pathophysiology of ODS, the cells mainly involved are the astrocytes. A recent *in vitro* study [26] has demonstrated early astrocytes death after rapid correction of hyponatremia. Astrocyte apoptosis is followed by the loss of the communication between astrocytes and oligodendrocytes, which is crucial for myelination processes [27,28]. In addition, following astrocytes death inflammatory responses are induced, such as *pro*-inflammatory cytokines production and microglia activation, eventually resulting in demyelination [26] (Figure 3).

## 3. From Pathophysiology to Clinical Practice

### 3.1. Hyponatremic Encephalopathy

Hyponatremic encephalopathy is defined as central nervous system dysfunction due to hyponatremia and occurs when brain fails in regulating its volume, such as in acute hyponatremia or when other risk factors are present [8]. The occurrence of death or permanent brain damage in association with symptomatic hyponatremia is well known and has been described since 70 years ago [29,30,31], but only in the last three decades it was clarified that hyponatremia itself can be lethal, independently of the underlying medical conditions [32,33,34,35,36].

Symptomatic hyponatremia (Table 1) develops primarily in a limited number of clinical settings. The most common condition in which hyponatremic encephalopathy occurs is in postoperative patients, with a prevalence of about 1% [33,37,38]. The most important preventable risk factor is the postoperative administration of intravenous hypotonic fluids [39]. Two other groups of patients who are at high risk to develop hyponatremic brain damage are menstruant women and prepuberal children [33,35]. Estrogens have been shown to impair the activity of the Na^+^-K^+^ ATPase pump in brain and in other tissues [40,41,42], thus reducing brain adaptation to hyponatremia, whereas androgens can enhance it [43,44]. Moreover, estrogens seem to regulate water movement by affecting the expression of AQP1 and AQP4 and by increasing circulating levels of ADH, which causes water retention, hyponatremia worsening and water influx into the brain [45]. Furthermore, it has been demonstrated that ADH causes vasoconstriction in female rat brain with a consequent cerebral hypoxia that impairs even more brain adaptation [46]. Prepuberal children are another group of high-risk patients because physical factors, such us a discrepancy between skull and brain size, make them more predisposed to cerebral injury in case of brain edema [8,35]. In addition, the pediatric brain has a reduced Na^+^-K^+^ ATPase activity compared to the adult brain [47], thus impairing adaptive extrusion of solutes during brain swelling. Finally, hypoxemia, which is another cause of the Na^+^-K^+^ ATPase system dysfunction, greatly increases the risk of death or permanent brain damage in symptomatic hyponatremia. In these patients an episode of hypoxia can result from other underlying conditions (e.g., congestive heart failure) or as a consequence of central respiratory depression or neurogenic pulmonary edema that are well-described complications of cerebral edema and brain erniation [8,48,49].

**Table 1:** Clinical features of hyponatremic encephalopathy.

Early	Nausea/Vomiting/Anorexia
Advanced	Headache
	Muscular cramps
	Weakness
	Lethargy
	Restlessness
	Disorientation
	Depressed reflexes
Far advanced	Seizures
	Coma
	Respiratory arrest

Severely symptomatic hyponatremia represents a medical emergency and a prompt correction is mandatory. In patients with hypervolemic and euvolemic hyponatremia, the first line therapy is hypertonic saline solution (3% NaCl) infusion, usually in association with furosemide to limit the expansion of the extracellular volume [7]. Most patients with hypovolemic hyponatremia can be treated successfully with isotonic saline solution (0.9% NaCl), but in the presence of severe symptoms, such as seizures or coma, hypertonic saline infusion is required [7]. The initial goal is to obtain a 1–2 mmol/L/h increase in serum [Na^+^] to a total of 4–6 mmol/L, which appears to be sufficient to reverse the most severe complications of acute hyponatremia [2]. After this initial correction, on going correction for hyponatremia should not exceed a serum [Na^+^] correction of 8–12 mmol/L/day or 18–24 mmol/L in the first 48 h, to avoid the risk of ODS [7,50].

### 3.2. Chronic Hyponatremia

Chronic hyponatremia has been traditionally defined as an asymptomatic condition because of the previously described adaptive mechanisms that counteract brain swelling. However, recent studies have demonstrated that this condition may also negatively affect the health status. In a case-control study it has been observed that mild chronic hyponatremia (serum [Na^+^] 126 ± 5 mEq/L, mean ± SE) was associated with gait impairment, attention deficit and increased risk of falls [10]. The hyponatremic patients included in the study were considered asymptomatic during the first clinical examination at the medical emergency department, but it was found that they had significant deficits and fell significantly more frequently than normonatremic controls (21.3% *vs.* 5.3%) when evaluated with specific gait and attention tests. In agreement with these observations, “fall” was frequently recorded as a reason for admission at the medical emergency department. The decrease of the attention capabilities and the gait instability observed in chronic hyponatremic patients might justify the increased risk of falls, but the underlying mechanisms are still to be elucidated. One of these mechanisms might be neurotransmitters loss, such as glutamate [18], as previously discussed. An improvement in the gait tests was recorded after correction of hyponatremia, suggesting that a proper treatment of these patients could prevent a considerable number of falls and hospitalizations. Another study demonstrated that chronic moderate hyponatremia impaired memory in rats and that the correction of the electrolyte imbalance with the vasopressin receptor antagonist tolvaptan could reduce this effect [51]. Vasopressin receptors antagonists or vaptans, a new class of drugs that promotes aquaresis causing a decrease in urine osmolality and an increase in serum [Na^+^], have been approved for the treatment of hypervolemic and euvolemic hyponatremia in USA and euvolemic hyponatremia in Europe [2,52]. It has been shown that tolvaptan, the only vaptan available for oral use, improves the Mental Component Summary of the SF-12 General Health Survey, thus suggesting that the correction of serum [Na^+^] may effectively counteract the reduced mental performances observed in hyponatremic patients [52]. Mild chronic hyponatremia has also been associated with detrimental effects on bone, in particular increased risk of osteoporosis and fractures, also independently of bone demineralization [53,54,55,56,57]. The increased bone fragility together with gait instability makes chronic hyponatremia a new risk factor for fractures, especially in the elderly, and suggests the importance to correct this disorder even when chronic and mild. The traditional treatment of apparently asymptomatic or mildly symptomatic euvolemic or hypervolemic hyponatremia is represented by fluid restriction, whereas in hypovolemic hyponatremia rehydration with isotonic saline solution remains the first choice therapy [2]. In those cases in which severe symptoms are present, hypertonic saline solution infusion can be recommended initially. Patients with chronic hyponatremia are exposed to the risk of ODS, and recently published recommendations have suggested that the correction rate should be 4–8 mmol/L/day in patients at low-moderate risk of ODS with a lower limit to 4–6 mmol/L/day in patients at high risk of ODS [2]. Vaptans represent a valid and effective alternative to correct mildly symptomatic or apparently asymptomatic hyponatremia as an alternative to fluid restriction, if the latter does not work or is not well tolerated, or moderately symptomatic hyponatremia as an alternative to hypertonic saline infusion [2,58].

### 3.3. Osmotic Demyelination Syndrome

ODS was recognized for the first time as a complication of an overly rapid correction of hyponatremia in 1976 [59]. The exact prevalence of this syndrome is not known but in large autopsy series it was estimated to be of 0.25%–0.50% in the general population and the majority of the cases were not diagnosed premortem [60,61].

The risk to develop ODS depends on several factors. First of all, the patients at risk to develop ODS are those with chronic hyponatremia, especially if starting serum [Na^+^] is <120 mmol/L, whereas in acute hyponatremia compensatory solute losses are not present and therefore ODS is unlikely to occur [2]. Other common risk factors are hypokalemia, alcoholism, malnutrition and advanced liver diseases including liver transplantation [2,60,61]. These conditions promote the occurrence of ODS because they are associated with alterations in cellular volume control, thus reducing the brain’s tolerance to an acute osmotic stress [60,61]. In particular, the correction of concomitant hypokalemia may cause a more rapid increase of serum [Na^+^] because the Na^+^-K^+^ ATPase extrudes sodium as potassium enters the cell to restore depleted intracellular potassium storage, thus increasing the correction rate of hyponatremia [60,61]. In some conditions, such as in chronic alcoholism, advanced liver disease, dialysis disequilibrium syndrome and after therapy for hyperammonemia, ODS can occur both in presence of, and without rapid correction of hyponatremia [60], suggesting that any change in extracellaular fluid osmolality, due to serum osmoles other than sodium, may cause myelinolysis.

The clinical presentation of ODS may vary and depends on the severity of the injury and the involved areas. The most frequent form is isolated central pontine myelinolysis, which occurs in about 50% of cases, whereas in 30% both pontine and extrapontine areas are involved and in 20% isolated extra-pontine myelinolysis occurs [62]. The most frequently involved sites, beside the pons, are cerebellum, extreme and external capsule, lateral geniculate body, putamen, ippocampus, thalamus, caudate nucleus and cerebral cortex [61]. Signs and symptoms of ODS include quadriparesis, dysarthria, dysphagia, and other pseudobulbar symptoms (Table 2). Usually, the development of these alterations occurs several days after the correction of hypoosmolality [63]. In some cases, as suggested from autopsy series, ODS may be asymptomatic or mildly symptomatic [62,64].

The diagnosis of ODS is clinical in patients with neurological symptoms after a recent overly rapid correction of hyponatremia, and can be confirmed by the presence of typical lesions at magnetic resonance imaging [65]. ODS prognosis was traditionally considered severe with an elevate mortality rate, ranging from 50% to 90% at three months from diagnosis [66,67]. However, a more recently published review of 44 German patients with ODS showed a 6% mortality rate and 40% of patients were discharged without severe neurological disease [68]. Because validated and effective therapies for ODS are lacking, preventing ODS occurrence appears to be of fundamental importance. The correction rate of hyponatremia should not exceed the recommended limits in patients at risk of ODS, as previously detailed. If overcorrection occurs, therapeutic re-lowering of serum [Na^+^] could be considered, especially in patients at high risk of ODS, even though validate trials in humans are not available [2,60,69,70]. Re-lowering of serum [Na^+^] can be achieved by administering desmopressin (2–5 mcg every 8 hours) and/or by infusion of 5% dextrose in water. Other experimental approaches consist of administration of high dose of corticosteroids, myoinositol or urea, but large studies in humans are lacking and controversial [2,60,71,72,73].

**Table 2:** Signs and symptoms of osmotic demyelination syndrome (ODS).

Impairment in Short-Term Memory
Attention deficit
Dysarthria
Dysphagia
Flaccid Quadriparesis
Oculomotor abnormalities
Ataxia
Mutism
Parkinsonism
Catatonia
Dystonia
Tremor
“Locked-in” syndrome
Seizures
Coma

## 4. New Perspectives in Chronic Hyponatremia: Beyond the “Osmotic Theory”

The recent demonstration of detrimental systemic effects secondary to chronic hyponatremia, traditionally defined as an asymptomatic or mildly symptomatic condition, opens a new scenario in understanding the pathophysiology of hyponatremia and its clinical consequences that may extend beyond the “osmotic theory”. In fact, the adaptive mechanisms that normally counteract cells swelling in chronic hyponatremia, do not explain in principle the presence of the neurological and extraneurological alterations observed also in this condition. An interesting hypothesis could be that hyponatremia directly affects the cellular homeostasis and hence the health status, independently of reduced osmolality. Recently, Barsony *et al.* demonstrated that sustained low extracellular [Na^+^] directly activated osteoclastogenesis and osteoclastic bone resorption in a murine model [56]. Interestingly, they found a [Na^+^]-dependent decrease in the activity of the vitamin C transporter, which resulted in a decrease of the uptake of ascorbic acid. Ascorbic acid not only has a central role in regulating the equilibrium between osteoblastic functions and osteoclastogenenesis [74,75], but it is also an essential defence against oxidative stress [75]. In the murine model of Barsony *et al.*, the reduction of ascorbic acid secondary to hyponatremia caused an increased production of free reactive oxygen species and stimulated several oxidative stress responses [56]. Oxidative stress is a well-known, common denominator in multiple pathologies related to senescence [76] and contributes to the aging process in the brain [77]. Therefore, a very interesting hypothesis might be that chronic hyponatremia plays a direct role in the pathogenesis of the degenerative processes, in particular those related to aging. Furthermore, the prevalence of hyponatremia increases progressively with aging, affecting up to 10% of patients living at home and 20% of nursing home residents [78] and the elderly represent the sub-population in which the effects of hyponatremia have a major impact in terms of disability, morbidity and mortality [1,3,10]. The link between chronic hyponatremia and senescence seems to be supported by the recent evidence that chronic hyponatremia exacerbated multiple manifestations of senescence, such as osteoporosis, hypogonadism, increased body fat and sarcopenia, in male rats [79]. In this new scenario, the understanding of the potential direct effects of chronic hyponatremia on the brain, which is one of the main targets of both hyponatremia and senescence, appears to be of particular interest. With regard to this point, we have recently demonstrated that low extracellular [Na^+^] directly produces detrimental effects, independently of reduced osmolality in an *in vitro* neuronal model of chronic hyponatremia [80]. We observed that sustained low extracellular [Na^+^], also in the presence of unaltered osmolality, causes cell distress, in particular reduced cell viability and adhesion, altered expression of anti-apoptotic genes and reduced ability to differentiate into a mature neuronal phenotype. A comprehensive microarray analysis showed that the most altered functions in the presence of low extracellular [Na^+^] appeared to be those involved in “cell death and survival”. Interestingly, the expression of the heme oxygenase (HMOX-1) gene showed the highest increase. HMOX-1 encodes an inducible stress protein involved in heme turnover [81], which exerts a potent antioxidant and anti-apoptotic activity in various cells, in particular in neurons [82]. In the brain, HMOX-1 expression increases in response to oxidative stress and protects neuronal cells from oxidative damage such as that caused by cerebral ischemia [83] or ethanol intoxication [84]. These findings are in agreement with those demonstrated by Barsony *et al.* [56] and reinforce the hypothesis that chronic hyponatremia may have a role in brain distress and aging. These effects are therefore, at least in part, directly related to reduced serum [Na^+^] through pathways that appear to involve mainly oxidative stress. Admittedly, these original results may be a key to understanding the presence of signs and symptoms in chronic hyponatremia despite the presence of adaptive mechanisms.

## 5. Conclusions

Hyponatremia is a very common electrolyte disorder, especially in the elderly. The consequences of hyponatremia, in particular when acute, on the brain may be clinically evident and severe, including permanent disability or death. An overly rapid correction of hyponatremia may also expose patients to dramatic consequences. Furthermore, it is necessary to also carefully consider chronic hyponatremia, apparently a benign condition, which has been found to be associated with systemic detrimental effects and with a senescence effect. Recent findings indicated that low sodium *per se* is able to disrupt cell homeostasis and this may explain some of the signs and symptoms described in mild chronic hyponatremia. Nevertheless, further studies are necessary to clearly elucidate the molecular mechanisms involved in the response to low sodium and their reversibility following correct therapeutic strategies.

## References

[1] Upadhyay A., Jaber B.L., Madias N.E. (2006). Incidence and prevalence of hyponatremia. Am. J. Med..

[2] Verbalis J.G., Goldsmith S.R., Greenberg A., Korzelius C., Schrier R.W., Sterns R.H., Thompson C.J. (2013). Diagnosis, evaluation, and treatment of hyponatremia: Expert panel recommendations. Am. J. Med..

[3] Corona G., Giuliani C., Parenti G., Norello D., Verbalis J.G., Forti G., Maggi M., Peri A. (2013). Moderate hyponatremia is associated with increased risk of mortality: Evidence from a meta-analysis. PLoS One.

[4] Kirkman M.A., Albert A.F., Ibrahim Q.A., Doberenz D. (2013). Hyponatremia and Brain Injury: Historical and Contemporary Perspectives. Neurocrit. Care.

[5] Rabinstein A.A., Wijdicks E.F. (2003). Hyponatremia in critically ill neurological patients. Neurologist.

[6] De Vita M.V., Gardenswartz M.H., Konecky A., Zabetakis P.M. (1990). Incidence and etiology of hyponatremia in an intensive care unit. Clin. Nephrol..

[7] Adrogué H.J., Madias N.E. (2000). Hyponatremia. N. Engl. J. Med..

[8] Ayus C., Achinger S.G., Arieff A. (2008). Brain cell volume regulation in hyponatremia: Role of sex, age, vasopressin, and hypoxia. Am. J. Physiol. Renal Physiol..

[9] Pasantes-Morales H., Franco R., Ordaz B., Ochoa L.D. (2002). Mechanisms Counteracting Swelling in Brain Cells During Hyponatremia. Arch. Med. Res..

[10] Renneboog B., Musch W., Vandemergel X., Manto M.U., Decaux G. (2006). Mild Chronic Hyponatremia is Associated with Falls, Unsteadiness, and Attention Deficits. Am. J. Med..

[11] Kimelberg H.K. (2004). Water homeostasis in the brain: Basic concepts. Neuroscience.

[12] Kimelberg H.K., Gilles R. (1991). Swelling and volume control in brain astroglial cells. Advances in Comparative and Environmental Physiology Series.

[13] Papadopoulos M.C., Verkman A.S. (2007). Aquaporin-4 and brain edema. Pediatr. Nephrol..

[14] Del Bigio M.R., Fedoroff S. (1990). Swelling of astroglia *in vitro* and the effect of arginine vasopressin and atrial natriuretic peptide. Acta Neurochir..

[15] Simard M., Nedergaard M. (2004). The neurobiology of glia in the context of water and ion homeostasis. Neuroscience.

[16] Okada Y., Sato K., Numata T. (2009). Pathophysiology and puzzles of the volume-sensitive outwardly rectifying anion channel. J. Physiol..

[17] Fisher S.K., Heacock A.M., Keep R.F., Foster D.J. (2010). Receptor regulation of osmolyte homeostasis in neural cells. J. Physiol..

[18] Verbalis J.G. (2010). Brain volume regulation in response to changes in osmolality. Neuroscience.

[19] Okada Y. (1997). Volume expansion-sensing outward-rectifier Cl-channel: Fresh start to the molecular identity and volume sensor. Am. J. Physiol..

[20] Sjoblom E., Hojer J., Ludwigs U. (1997). Fatal hyponatraemic brain oedema due to common gastroenteritis with accidental water intoxication. Intensive Care Med..

[21] Sterns R.H. (1987). Severe symptomatic hyponatremia: Treatment and outcome. A study of 64 cases. Ann. Intern. Med..

[22] Lien Y.H., Shapiro J.I., Chan L. (1991). Study of brain electrolytes and organic osmolytes during correction of chronic hyponatremia. Implications for the pathogenesis of central pontine myelinolysis. J. Clin. Investig..

[23] Verbalis J.G., Gullans S.R. (1993). Rapid correction of hyponatremia produces differential effects on brain osmolyte and electrolyte reaccumulation in rats. Brain Res..

[24] Berl T. (1990). Treating hyponatremia: Damned if we do and damned if we don’t. Kidney Int..

[25] Brown W.D. (2000). Osmotic demyelination disorders: Central pontine and extrapontine myelinolysis. Curr. Opin. Neurol..

[26] Gankam Kengne F., Nicaise C., Soupart A., Boom A., Schiettecatte J., Pochet R., Brion J.P., Decaux G. (2011). Astrocytes are an early target in osmotic demyelination syndrome. J. Am. Soc. Nephrol..

[27] Nagy J.I., Rash J.E. (2000). Connexins and gap junctions of astrocytes and oligodendrocytes in the CNS. Brain Res. Rev..

[28] Rouach N., Koulakoff A., Abudara V., Willecke K., Giaume C. (2008). Astroglial metabolic networks sustain hippocampal synaptic transmission. Science.

[29] Arieff A.I., Llach F., Massry S.G., Kerian A. (1976). Neurological manifestations and morbidity of hyponatremia: Correlation with brain water and electrolytes. Medicine.

[30] Helwig F.C., Schultz C.B., Curry D.E. (1935). Water intoxication: Report of a fatal human case, with clinical, pathologic and experimental studies. JAMA.

[31] Zimmermann B., Wangensteen O.H. (1952). Observations on water intoxication in surgical patients. Surgery.

[32] Arieff A.I. (1986). Hyponatremia, convulsions, respiratory arrest, and permanent brain damage after elective surgery in healthy women. N. Engl. J .Med..

[33] Ayus J.C., Wheeler J.M., Arieff A.I. (1992). Postoperative hyponatremic encephalopathy in menstruant women. Ann. Intern. Med..

[34] Baggish M.S., Brill A.I., Rosenweig B. (1993). Fatal acute glycine and sorbitol toxicity during operative hysteroscopy. J. Gynecol. Surg..

[35] Arieff A.I., Ayus J.C., Fraser C.L. (1992). Hyponatraemia and death or permanent brain damage in healthy children. BMJ.

[36] Fenves A.Z., Thomas S., Knochel J.P. (1996). Beer potomania: Two cases and review of the literature. Clin. Nephrol..

[37] Baran D., Hutchinson T.A. (1984). The outcome of hyponatremia in a general hospital population. Clin. Nephrol..

[38] Anderson R.J., Chung H.M., Kluge R., Schrier R.W. (1985). Hyponatremia: A prospective analysis of its epidemiology and the pathogenetic role of vasopressin. Ann. Intern. Med..

[39] Arieff A. (2001). Treatment of hyponatremic encephalopathy with antagonists to antidiuretic hormone. J. Lab. Clin. Med..

[40] Futo J., Shay J., Block S., Holt J., Beach M., Moss J. (1992). Estrogen and progesterone withdrawal increases cerebral vasoreactivity to serotonin in rabbit basilar artery. Life Sci..

[41] Sarrel P.M., Lufkin E.G., Oursler M.J., Keefe D. (1994). Estrogen actions in arteries, bone, and brain. Sci. Am. Sci. Med..

[42] Fraser C.L., Swanson R.A. (1994). Female sex hormones inhibit volume regulation in rat brain astrocyte culture. Am. J. Physiol..

[43] Fraser C.L., Sarnacki P. (1989). Na^+^-K^+^ ATPase pump function in rat brain synaptosomes is different in males and females. Am. J. Physiol. Endocrinol. Metab..

[44] Guerra M., del Castillo A.R., Battaner E., Mas M. (1987). Androgens stimulate preoptic area Na^+^-K^+^ ATPase activity in male rats. Neurosci. Lett..

[45] Fraser C.L., Arieff A.I. (1997). Epidemiology, Pathophysiology, and Management of Hyponatremic Encephalopathy. Am. J. Med..

[46] Kozniewska E., Roberts T.P.L., Vexler Z.S., Oseka M., Kucharczyk J., Arieff A.I. (1995). Hormonal dependence of the effects of metabolic encephalopathy on cerebral perfusion and oxygen utilization in the rat. Circ. Res..

[47] Halberthal M., Halperin M.L., Bohn D. (2001). Acute hyponatremia in children admitted to hospital: Retrospective analysis of factors contributing to its development and resolution. Br. Med. J..

[48] Ayus J.C., Arieff A.I. (1995). Pulmonary complications of hyponatremic encephalopathy: Noncardiogenic pulmonary edema and hypercapnic respiratory failure. Chest.

[49] Filippatos T.D., Elisaf M.S. (2013). Hyponatremia in patients with heart failure. World J. Cardiol..

[50] Adrogué H.J. (2005). Consequences of inadequate management of hyponatremia. Am. J. Nephrol..

[51] Miyazaki T., Ohmoto K., Hirose T., Fujiki H. (2010). Chronic hyponatremia impairs memory in rats: Effects of vasopressin antagonist tolvaptan. J. Endocrinol..

[52] Schrier R.W., Gross P., Gheorghiade M., Berl T., Verbalis J.G., Czerwiec F.S., Orlandi C., SALT Investigators (2006). Tolvaptan, a selective oral vasopressin V_2_-receptor antagonist, for hyponatremia. N. Engl. J. Med..

[53] Gankam K.F., Andres C., Sattar L., Melot C., Decaux G. (2008). Mild hyponatremia and risk of fracture in the ambulatory elderly. QJM.

[54] Kinsella S., Moran S., Sullivan M.O., Molloy M.G., Eustace J.A. (2010). Hyponatremia independent of osteoporosis is associated with fracture occurrence. Clin. J. Am. Soc. Nephrol..

[55] Verbalis J.G., Barsony J., Sugimura Y., Tian Y., Adams D.J., Carter E.A., Resnick H.E. (2010). Hyponatremia-induced osteoporosis. J. Bone Miner. Res..

[56] Barsony J., Sugimura Y., Verbalis J.G. (2011). Osteoclast response to low extracellular sodium and the mechanism of hyponatremia-induced bone loss. J. Biol. Chem..

[57] Hoorn E.J., Rivadeneira F., van Meurs J.B., Ziere G., Stricker B.H., Hofman A., Pols H.A., Zietse R., Uitterlinden A.G., Zillikens M.C. (2011). Mild hyponatremia as a risk factor for fractures: The Rotterdam Study. J. Bone Miner. Res..

[58] Peri A. (2013). Clinical review: The use of vaptans in clinical endocrinology. J. Clin. Endocrinol. Metab..

[59] Tomlinson B.E., Pierides A.M., Bradley W.G. (1976). Central pontine myelinolysis. Two cases with associated electrolyte disturbance. Q. J. Med..

[60] King J.D., Rosner M.H. (2010). Osmotic demyelination syndrome. Am. J. Med. Sci..

[61] Corona G., Simonetti L., Giuliani C., Sforza A., Peri A. (2014). A case of osmotic demyelination syndrome occurred after the correction of severe hyponatraemia in hyperemesis gravidarum. BMC Endocr. Disord..

[62] Wright D.G., Laureno R., Victor M. (1979). Pontine and extrapontine myelinolysis. Brain.

[63] Kleinschmidt-Demasters B.K., Rojiani A.M., Filley C.M. (2006). Central and extrapontine myelinolysis: Then and now. J. Neuropathol. Exp. Neurol..

[64] Newell K.L., Kleinschmidt-DeMasters B.K. (1996). Central pontine myelinolysis at autopsy: A twelve year retrospective analysis. J. Neurol. Sci..

[65] Miller G.M., Baker H.L., Okazaki H. (1988). Central pontine myelinolysis and its imitators: MR findings. Radiology.

[66] Gocht A., Colmant H.J. (1987). Central pontine and extrapontine myelinolysis: A report of 58 cases. Clin. Neuropathol..

[67] McCormick W.F., Danneel C.M. (1967). Central pontine myelinolysis. Arch. Intern. Med..

[68] Menger H., Jorg J. (1999). Outcome of central pontine and extrapontine myelinolysis (*n* = 44). J. Neurol..

[69] Oya S., Tsutsumi K., Ueki K. (2001). Reinduction of hyponatremia to treat central pontine myelinolysis. Neurology.

[70] Soupart A., Ngassa M., Decaux G. (1999). Therapeutic relowering of the serum sodium in a patient after excessive correction of hyponatremia. Clin. Nephrol..

[71] Sterns R.H., Silver S.M. (2006). Brain volume regulation in response to hypoosmolality and its correction. Am. J. Med..

[72] Decaux G., Soupart A. (2003). Treatment of symptomatic hyponatremia. Am. J. Med. Sci..

[73] Perianayagam A., Sterns R.H., Silver S.M. (2008). DDAVP is effective in preventing and reversing inadvertent overcorrection of hyponatremia. Clin. J. Am. Soc. Nephrol..

[74] Wu X., Itoh N., Taniguchi T., Nakanishi T., Tatsu Y., Yumoto N., Tanaka K. (2003). Zinc-induced sodium-dependent vitamin C transporter 2 expression: Potent roles in osteoblast differentiation. Arch. Biochem. Biophys..

[75] Xiao X.H., Liao E.Y., Zhou H.D., Dai R.C., Yuan L.Q., Wu X.P. (2005). Ascorbic acid inhibits osteoclastogenesis of RAW264.7 cells induced by receptor activated nuclear factor kappaB ligand (RANKL) *in vitro*. J. Endocrinol. Investig..

[76] Sohal R.S., Weindruch R. (1996). Oxidative stress, caloric restriction, and aging. Science.

[77] Yakner B.A., Lu T., Loerch P. (2008). The Aging Brain. Annu. Rev. Pathol..

[78] Miller M., Morley J.E., Rubenstein L.Z. (1995). Hyponatremia in a nursing home population. Am. Geriatr. Soc..

[79] Barsony J., Manigrasso M.B., Xu Q., Tam H., Verbalis J.G. (2013). Chronic hyponatremia exacerbates multiple manifestations of senescence in male rats. Age (Dordr.).

[80] Benvenuti S., Deledda C., Luciani P., Modi G., Bossio A., Giuliani C., Fibbi B., Peri A. (2013). Low extracellular sodium causes neuronal distress independently of reduced osmolality in an experimental model of chronic hyponatremia. Neuromol. Med..

[81] Mancuso C. (2004). Heme oxygenase and its products in the nervous system. Antioxid. Redox Signal..

[82] Chen K., Gunter K., Maines M.D. (2000). Neurons overexpressing heme oxygenase-1 resist oxidative stress-mediated cell death. J. Neurochem..

[83] Takizawa S., Hirabayashi H., Matsushima K., Tokuoka K., Shinohara Y. (1998). Induction of heme oxygenase protein protects neurons in cortex and striatum, but not in hippocampus, against transient forebrain ischemia. J. Cereb. Blood Flow Metab..

[84] Ku B.M., Joo Y., Mun J., Roh G.S., Kang S.S., Cho G.J. (2006). Heme oxygenase protects hippocampal neurons from ethanol-induced neurotoxicity. Neurosci. Lett..

